# Nutritional ketosis delays the onset of isoflurane induced anesthesia

**DOI:** 10.1186/s12871-018-0554-0

**Published:** 2018-07-18

**Authors:** Csilla Ari, Zsolt Kovács, Cem Murdun, Andrew P. Koutnik, Craig R. Goldhagen, Christopher Rogers, David Diamond, Dominic P. D’Agostino

**Affiliations:** 1 Department of Psychology, Hyperbaric Neuroscience Research Laboratory, University of South Florida, 4202 East Fowler Ave, PCD3127, Tampa, FL 33620 USA; 2 Department of Molecular Pharmacology and Physiology, Laboratory of Metabolic Medicine, Morsani College of Medicine, University of South Florida, 12901 Bruce B. Downs Blvd, Tampa, FL 33612 USA; 3 Savaria Department of Biology, ELTE Eötvös Loránd University, Savaria Campus, Károlyi Gáspár tér 4, Szombathely, Hungary

**Keywords:** Latency, Anesthetic induction, Isoflurane, Ketogenic diet, Exogenous ketogenic supplements, Ketosis, Rodent models, Anesthesia, Ketones

## Abstract

**Background:**

Ketogenic diet (KD) and exogenous ketone supplements can evoke sustained ketosis, which may modulate sleep and sleep-like effects. However, no studies have been published examining the effect of ketosis on the onset of general isoflurane induced anesthesia. Therefore, we investigated the effect of the KD and different exogenous ketogenic supplements on the onset of akinesia induced by inhalation of isoflurane.

**Methods:**

We used a high fat, medium protein and low carbohydrate diet (KD) chronically (10 weeks) in the glucose transporter 1 (GLUT1) deficiency (G1D) syndrome mice model and sub-chronically (7 days) in Sprague-Dawley (SPD) rats. To investigate the effect of exogenous ketone supplements on anesthetic induction we also provided either 1) a standard rodent chow diet (SD) mixed with 20% ketone salt supplement (KS), or 2) SD mixed with 20% ketone ester supplement (KE; 1,3 butanediol-acetoacetate diester) to G1D mice for 10 weeks. Additionally, SPD rats and Wistar Albino Glaxo Rijswijk (WAG/Rij) rats were fed the SD, which was supplemented by oral gavage of KS or KE for 7 days (SPD rats: 5 g/kg body weight/day; WAG/Rij rats: 2.5 g/kg body weight/day). After these treatments (10 weeks for the mice, and 7 days for the rats) isoflurane (3%) was administered in an anesthesia chamber, and the time until anesthetic induction (time to immobility) was measured. Blood ketone levels were measured after anesthetic induction and correlation was calculated for blood beta-hydroxybutyrate (βHB) and anesthesia latency.

**Results:**

Both KD and exogenous ketone supplementation increased blood ketone levels and delayed the onset of isoflurane-induced immobility in all investigated rodent models, showing positive correlation between the two measurements. These results demonstrate that elevated blood ketone levels by either KD or exogenous ketones delayed the onset of isoflurane-induced anesthesia in these animal models.

**Conclusions:**

These findings suggest that ketone levels might affect surgical anesthetic needs, or could potentially decrease or delay effects of other narcotic gases.

## Background

Therapeutic ketosis has been proven to be neuroprotective. Ketosis improves symptoms of Alzheimer’s disease, Parkinson’s disease, schizophrenia, amyotrophic lateral sclerosis, glucose transporter 1 (GLUT1)-deficiency syndrome, autism, anxiety, depression, cancer, and epilepsy in patients and/or animal models [1–7]. The common problem with these disorders is that the cellular energetic status is compromised. The primary fuel source in a typical western (carbohydrate-based) diet is glucose, which is metabolized to yield acetyl-CoA, driving the citric acid cycle to produce adenosine triphosphate (ATP) and other intermediates associated with neurotransmitter and energy balance. However, in the absence of dietary glucose, the liver mobilizes fatty acids for fuel, generating ketone bodies, such as beta-hydroxybutyrate (βHB) and acetoacetate, which can be transported to other tissues in the body [8–10]. The ketone bodies are then converted back to acetyl-CoA in the mitochondria, allowing the citric acid cycle to continue generating ATP to meet energy demands. Importantly, ketone bodies are able to cross the blood-brain barrier and provide fuel to the brain when dietary glucose is insufficient [8, 10]. An increase in ketone bodies, called ketosis, can be achieved with either a ketogenic diet (KD) or exogenous ketones [3–5, 10]. A state of ketosis enables cells to function efficiently by using both ketones and glucose.

It is unknown whether therapeutic ketosis would protect the nervous system from external harmful substances, such as toxic gases. Isoflurane (1-chloro-2,2,2-trifluoroethyl difluoromethyl ether) has been used as an inhalational anesthetic in patients for about 45 years [11]. However, it’s mechanism of action remains unknown. Although anesthesia and naturally occurring sleep are considered different states, it is a widely accepted theory that inhalational anesthetics may exert their anesthetic effects via endogenous neural circuitries and brain areas (e.g., cerebral cortex and/or ventrolateral preoptic area containing predominately sleep active, GABAergic and galaninergic neurons), which are also implicated in the modulation of naturally occurring sleep, generating a sleep-like state [12–15].

KD and exogenous ketone supplements can evoke sustained ketosis [2, 5], potentially modulating sleep and sleep-like effects [16–19]. No studies have been published on how ketosis might affect the onset of general isoflurane induced anesthesia. Based on anecdotal information, GLUT1 deficiency (G1D) syndrome patients -while on strict KD in order to elevate their blood ketone levels, since their brain is not able to use glucose as fuel-, have experienced delayed onset of anesthesia (personal communication with GLUT1D Foundation). In the present study we investigated whether nutritional ketosis induced by different methods can modulate the onset of isoflurane-induced anesthesia in animal models with and without pathology [20, 21]. Our methods included inducing ketosis in the animals sub-chronically (1 week) or chronically (10 weeks) either by ketogenic diet or with standard diet supplemented with ketone supplements. To investigate how animal models of different species and strains respond to isoflurane-evoked anesthetic induction while consuming the ketogenic diet or exogenous ketone supplements, we used one species of animal model without pathology and two species with pathology. Sprague-Dawley (SPD) rats do not have pathology, while Wistar Albino Glaxo Rijswijk (WAG/Rij) rats are a model of human absence epilepsy and G1D mice are a mouse model of GLUT1 deficiency syndrome. We investigated the time until anesthetic induction, defined as the onset of immobility, a widely used measure of anesthetic potency [22] after administering 3% isoflurane.

This work is potentially clinically relevant since a ketosis-induced change in the latency to anesthesia may need to be considered when patients are undergoing anesthetic procedures (e.g., increased time prior to loss of consciousness before a medical procedure). It is also potentially relevant that ketosis-induced delay in the onset of anesthesia may indicate that ketosis may potentially also protects the nervous system from other, harmful gases.

We hypothesized that nutritional ketosis evoked by KD or exogenous ketogenic supplements would delay the latency to onset of anesthesia. Here, we demonstrated that in all tested animal models the KD and both exogenous ketone supplements (ketone ester and ketone salt) did indeed delay the onset of isoflurane anesthesia, likely due to the neuroprotective properties of ketones.

## Methods

### Animals

Animal treatments were carried out according to the University of South Florida Institutional Animal Care and Use Committee (IACUC) guidelines (Protocol #00001749 and # 00000457), Hungarian Act of Animal Care and Experimentation (1998, XXVIII, section 243), European Communities Council Directive 24 November 1986 (86/609/EEC) and EU Directive 2010/63/EU to use and treat animals in experimental laboratories. The experimental design was approved by the Animal Care and Experimentation Committee of the Eötvös Loránd University (Savaria Campus) and National Scientific Ethical Committee on Animal Experimentation (Hungary) under license number VA/ÉBNTF02/85–8/2016.

G1D male mice (*n* = 33; 3–5 months old, 17–27 g), SPD male rats (*n* = 45; 5–6 months old, 320–360 g) and WAG/Rij male rats (*n* = 24; 6 months old, 320–360 g) were kept under standard laboratory conditions (12:12 h light-dark cycle, light was on from 08.00 AM to 08.00 PM; free access to food and water; air-conditioned room at 22 ± 2 °C). All efforts were made to minimize pain and suffering and to reduce the number of animals used.

### Treatment administration and detection of immobility

In order to induce ketosis we administered either chronic (10 weeks) or sub-chronic (7 days) treatments. G1D mice were chronically fed by a standard rodent chow diet (SD/control; 2018 Teklad Global 18% Protein Rodent Diet, Harlan; *n* = 10), ketogenic diet (*n* = 5, KD) (Table 1), SD mixed with 20% KE (1,3 butanediol-acetoacetate diester) supplement (*n* = 11; KE) or SD mixed with 20% KS (Na^+^/K^+^ − βHB mineral salt) supplement (*n* = 7; KS) for 10 weeks.

**Table 1:** Macronutrient ratios of rodent standard diet and ketogenic diet used

Macronutrient Information	Standard Diet (SD)	Ketogenic Diet (KD)
% Cal from Fat	18.0	77.1
% Cal from Protein	24.0	22.4
% Cal from Carbohydrate	58.0	0.5
Caloric Density (Kcal/g)	3.1	4.7

Sprague-Dawley rats were fed with SD and gavaged with water (SD/control; *n* = 12), fed with KD and gavaged with water (n = 11, KD) or fed with SD and gavaged with KE (n = 11, KE) or KS (n = 11, KS) by intragastric gavage for 7 days.

WAG/Rij rats were also fed with standard diet and gavaged orally either with water (SD/ control; *n* = 8), with KE (n = 8, KE), or with KS (n = 8, KS) for 7 days. To familiarize the animals to the intragastric gavage method, exogenous ketone supplement gavage was preceded by water gavage for 5 days (adaptation period). Following the adaptation period, we administered well-tolerated doses of exogenous ketone supplements (SPD rats: 5 g/kg body weight/day KE and KS; WAG/Rij rats: 2.5 g/kg body weight/day KE and KS) by oral gavage [4, 5, 7]. After 10 weeks (for the mice) or 7 days treatments (for the rats), anesthesia was induced in an air tight anesthesia chamber with 3% isoflurane gas mixed with air. Immobility, time from chamber closure until end phase of anesthetic induction was measured and analyzed by a blinded observer via video recordings.

### Measurement of blood βHB levels

Blood was taken from the tail vein of G1D mice and from the saphenous vein of rats. βHB levels were measured by a commercially available glucose and ketone monitoring system (Precision Xtra™, Abbott Laboratories, Abbott Park, IL, USA) [4]. Baseline ketone levels were measured on the last (5th) day of the adaptation period (for rats) or before the chronic treatment started (for mice). Blood was collected and βHB levels were measured again after the last day of KD or ketone supplementation at ~ 10 min after the detection of immobility induced by isoflurane.

### Statistics

All data are presented as the mean ± standard error of the mean (S.E.M.). We measured and compared the latency of isoflurane-induced immobility in control and treatment groups, and compared baseline and final βHB levels. Data analysis was performed using GraphPad Prism version 6.0a using a two-way ANOVA with Tukey’s multiple comparisons test and unpaired t-test. Pearson correlation was calculated for blood βHB and anesthesia latency as individual data points (except in G1D mice, when the quality of the video recording did not allow the identification of individual mice, only the treatment group) and as group means. Results were considered significant when *p* < 0.05.

## Results

### Delayed anesthesia induction and elevated blood ketone levels in Sprague-Dawley rats

**Fig. 1:**
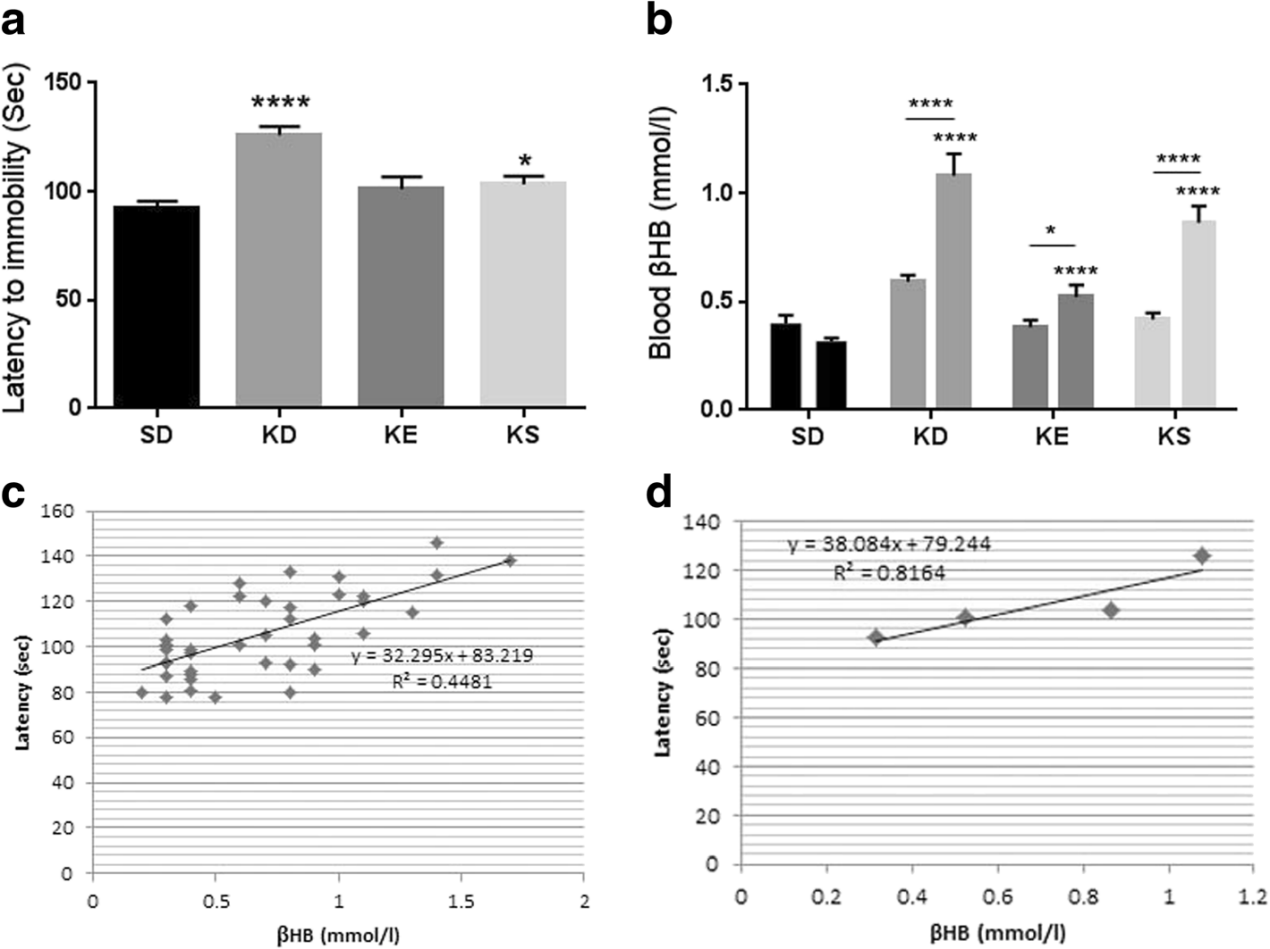
**a.** Latency to anesthesia induction measured by the time until immobility in Sprague-Dawley rats. In ketogenic diet (KD) and KS groups the latency to anesthesia was significantly longer (*p* < 0.0001 and *p* = 0.0337, respectively) compared to control (standard diet, SD); **b.** Blood βHB level was significantly elevated in all treatment groups, compared to control (ketogenic diet, KD: *p* < 0.0001, KE: *p* < 0.0001, KS: *p* < 0.0001) and compared to their baseline (ketogenic diet, KD: *p* = 0.0001, KE: *p* = 0.03, KS: *p* < 0.0001; interaction: F_3,80_ = 14.12, *p* < 0.0001; time: F_1,80=_ 45.75, *p* < 0.0001; treatment: F_3,80=_ 33.6, *p* < 0.0001). Bar on left represents baseline value, bar on the right represents value after treatment in each group; **c.** There was a positive correlation between latency to anesthesia induction and blood βHB levels when all individual data point was considered (R^2^ = 0.4481); **d.** There was a strong positive correlation between latency to anesthesia induction and blood βHB levels when the group means were considered (R^2^ = 0.8164)

Treatment with KD and SD with KS caused a significant increase in the number of seconds required before anesthetic induction (the time until immobility) (*p* < 0.0001, and *p* = 0.0337, respectively), compared to SD fed controls (Fig. 1a).

Rats in KD, KS and KE groups also exhibited a significant increase in the blood levels of βHB, compared to both control (*p* < 0.0001 for KD, KS and KE) and baseline (*p* < 0.0001 for KD and KS; p = 0.03 for KE; interaction: F_3,80_ = 14.12, *p* < 0.0001; time: F_1,80=_ 45.75, *p* < 0.0001; treatment: F_3,80=_ 33.6, *p* < 0.0001; Fig. 1b). The number of seconds required before the onset of anesthetic induction positively correlated with blood βHB levels when individual data points (R^2^ = 0.4481, *p* < 0.0001, Fig. 1c) or positively correlated, but not significantly when the group means were considered (R^2^ = 0.8164, *p* = 0.096) in SPD rats (Fig. 1d).

### Delayed anesthesia induction and elevated blood ketone levels in G1D mice

**Fig. 2:**
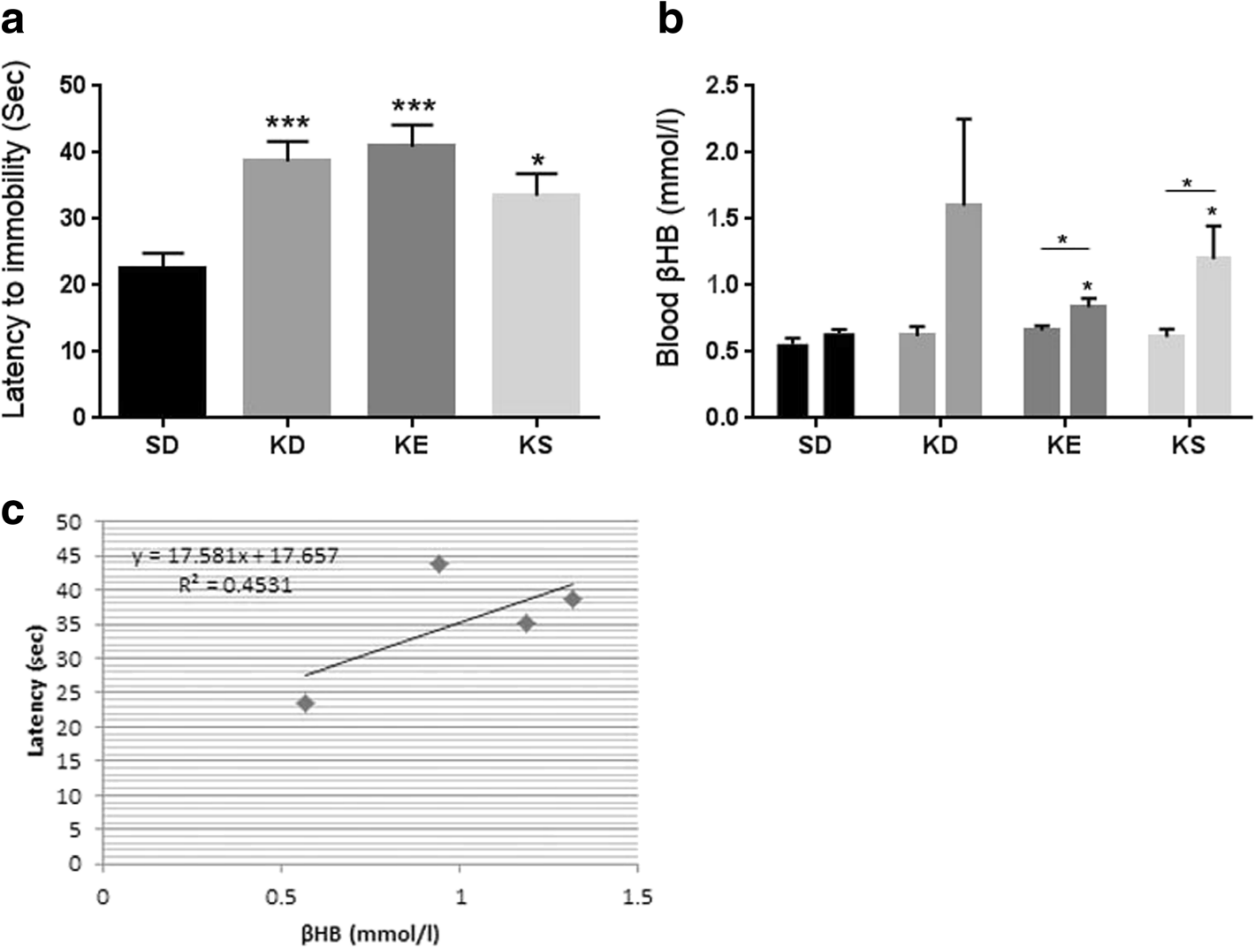
**a.** Latency to anesthesia induction measured by the time until immobility in G1D mice. In ketogenic diet (KD), KE and KS groups the latency to anesthesia was significantly longer (*p* = 0.0003, p = 0.0003, *p* = 0.0136, respectively) compared to control (standard diet, SD); **b.** Blood βHB level was significantly elevated in KE and KS groups, compared to control (*p* = 0.0117, *p* = 0.0169, respectively) and compared to their baseline (*p* = 0.02, *p* = 0.04, respectively). Bar on left represents baseline value, bar on the right represents value after treatment (after 10 weeks) in each group; **c.** There was a positive correlation between latency to anesthesia induction and blood βHB levels when the groups means were considered (R^2^ = 0.4531)

Chronic administration of KD, SD with KE, and SD with KS resulted in a significant increase in the time required for anesthetic induction (*p* = 0.0003, p = 0.0003, *p* = 0.0136, respectively), relative to control mice fed a SD (Fig. 2a).

The mice fed KD, or SD with KE, or KS exhibited elevated (KD: *p* = 0.07) or significantly increased (KE: *p* = 0.0117; KS: *p* = 0.0169) blood levels of βHB relative to control mice (Fig. 2b), confirming a state of ketosis. Significantly increased blood βHB levels were also detected in KE and KS supplemented mice, compared to their baseline levels (KE: *p* = 0.02; KS: *p* = 0.04). The increase in time required for induction of anesthesia positively correlated (not significantly) with the increase in blood levels of βHB when the group means were considered (R^2^ = 0.4531, *p* = 0.27) (Fig. 2c).

Delayed anesthesia induction and elevated blood ketone levels in WAG/Rij rats

**Fig. 3:**
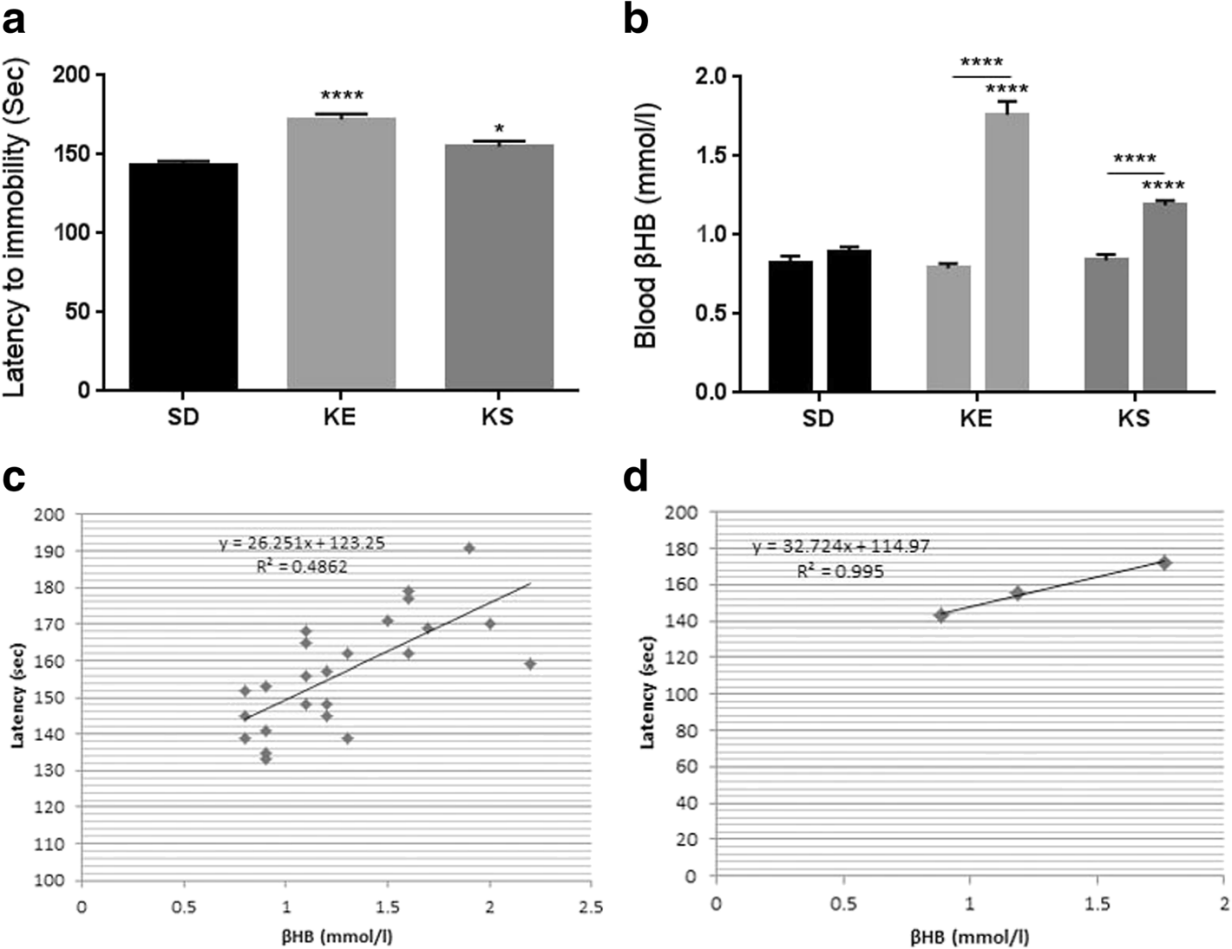
**a.** Latency to anesthesia induction measured by the time until immobility in WAG/Rij rats. In KE and KS groups the latency to anesthesia was significantly longer (*p* < 0.0001, *p* = 0.02, respectively) compared to control (standard diet, SD); **b.** Blood βHB level was significantly elevated in KE and KS groups, compared to control (*p* < 0.0001, *p* < 0.0001, respectively) and compared to their baseline (*p* < 0.0001, *p* < 0.0001, respectively; interaction: F_2,21_ = 51.23, *p* < 0.0001; time: F_1,21_ = 151, *p* < 0.0001; treatment: F_2,21_ = 37.44, *p* < 0.0001). Bar on left represents baseline value, bar on the right represents value after treatment in each group; **c.** There was a positive correlation between latency to anesthesia induction and blood βHB levels when all data point was considered (R^2^ = 0.4862); **d.** There was a strong positive correlation between latency to anesthesia induction and blood βHB levels when the group means were considered (R^2^ = 0.995)

SD with KE or KS significantly increased the time required before anesthetic induction (*p* < 0.0001, p = 0.02, respectively), compared to controls (SD) in WAG/Rij rats (Fig. 3a).

A significant increase in blood levels of βHB was demonstrated after the final treatment by KE and KS compared to both control (*p* < 0.0001 for both KE and KS) and baseline (*p* < 0.0001 for both KE and KS) levels (interaction: F_2,21_ = 51.23, *p* < 0.0001; time: F_1,21_ = 151, *p* < 0.0001; treatment: F_2,21_ = 37.44, *p* < 0.0001; Fig. 3b). Exogenous ketogenic supplements (KE and KS) evoked a significant increase in the number of seconds required before the onset of anesthetic induction, which - similar to both SPD rats and G1D mice (Figs. 1c,d and 2c) - positively correlated with blood βHB levels when individual data points (R^2^ = 0.4862, *p* = 0.0002, Fig. 3c) or the group means were considered (R^2^ = 0.995, *p* = 0.04) (Fig. 3d).

## Discussion

Overall, each rodent model studied here (G1D mice, SPD rats and WAG/Rij rats) that received either a KD or a standard diet with exogenous ketone supplements exhibited elevated blood ketone levels and delayed onset of isoflurane-induced immobility.

General anesthesia involves multiple features, including amnesia, unconsciousness, analgesia and immobility [22]. The basis of anesthesia actions, produced for example by inhalational anesthetics such as isoflurane, is likely to involve modulation of different molecular and cellular targets of local, peripheral and central pathways [11]. Even though isoflurane is widely used in clinical practice, its exact mechanism and sites of action remain largely unknown. However, it was demonstrated that the main targets for anesthetic effect of isoflurane are the GABA_A_ and NMDA receptors which generate neuronal inhibition and synaptic plasticity, respectively [15, 22–24]. Moreover other receptors and ion channels are also implicated in isoflurane-evoked modulatory effects (e.g. hyperpolarization/inhibition, reduced bursting frequency of neurons, and decrease in neurotransmitter release), such as glycine receptors, ATP-sensitive potassium (K_ATP_) channels, two-pore-domain ‘leak’ K^+^ channels, voltage gated Na^+^ channels, hyperpolarization-activated cyclic nucleotide-gated (HCN) channels, and Ca^2+^ channels [11, 22, 25, 26]. By means of these receptors and ion channels, isoflurane may suppress excitatory synaptic transmission (e.g., by decrease in glutamate release) or/and may enhance inhibitory mechanisms/net inhibitory current, which processes may result in immobilization by depression of reflex pathways of the spinal cord [22].

Although physiological differences have been demonstrated between general anesthesia-induced sleep-like state and naturally occurring sleep, studies suggest that similar brain nuclei, neuronal networks, signaling pathways and neurotransmitters may be implicated in both processes [13, 15]. For example, a specific hypothalamic nucleus (ventrolateral preoptic area) of sleep-active neurons participates not only in sleep promotion, but also in the anesthetic effect of isoflurane: isoflurane increased depolarization and the firing rate of sleep-active neurons in this brain area by reduction of basal potassium conductance [12–14]. On the contrary, other authors suggest that isoflurane may exert its anesthetic influence by direct inhibition of the arousal system, neurons of motor systems and cerebral cortex rather than by indirect mechanisms (e.g., potentiation of sleep-active neurons of ventrolateral preoptic area) [27].

The administration of the KD and exogenous ketone supplements results in ketosis (i.e., increase in βHB level), potentially leads to changes in sleep structure and time (e.g., decrease in total sleep time and increase in rapid eye movement/REM sleep time), which may be modulated, among others, via the ventrolateral preoptic area [4, 13, 18, 19]. As previous studies demonstrate the concentration, utilization and metabolism of ketone bodies may occur in a brain-region dependent manner [10, 16, 28]. As ketosis increases extracellular adenosine levels, as a consequence, it may evoke changes in neuronal activity by its receptors [17, 29]. According to previous studies adenosine also accumulates under certain conditions, such as sleep deprivation [13, 30]. In addition, the distribution of the adenosinergic system is uneven in the brain suggesting its different physiological/functional roles in different brain areas [29, 31]. Therefore, it is possible that adenosine, as an endogenous homeostatic factor in sleep regulation, may be a link between the ketosis induced anesthetic delay. It has been demonstrated that adenosine increased the excitability of ventrolateral preoptic area neurons by disinhibition [32], a mechanism, by which adenosine may promote its sleep-eliciting processes. Increased level of adenosine may decrease the release of excitatory neurotransmitter glutamate in the cerebral cortex and arousal system via inhibitory A_1_ type adenosine receptors (A_1_Rs) [27, 33] whereby adenosine may facilitate the anesthetic effects of isoflurane. Nevertheless, not only A_1_Rs, but also excitatory A_2A_ type adenosine receptors (A_2A_Rs) are implicated in sleep-wake regulation, which A_2A_Rs may increase the level of excitatory neurotransmitters in the brain [17, 29, 34] and may modify the anesthetic effects of isoflurane. As the influence of adenosine receptors on sleep-wake cycle is brain region-dependent (e.g., activation of A_1_Rs may generate both sleep and wakefulness depending on affected brain areas) [34] it is possible that KD and exogenous ketone supplementation may moderate the anesthetic effect of isoflurane by adenosine and its receptors. Based on these results, we can hypothesize that ketogenic diet and exogenous ketogenic supplements caused a delay in the onset of isoflurane-induced immobility possibly involving changes in adenosine-generated modulatory effects in different brain areas implicated in anesthetic effects.

The role of other neurotransmitter systems (e.g., GABAergic system), ion channels (e.g., K_ATP_ and Ca^2+^ channels) and other mechanisms of action of both isoflurane and ketosis/ketone bodies (e.g., bioenergetics or/mitochondrial changes) has been assumed in KD- and exogenous ketone supplementation-evoked influences on isoflurane-generated anesthetic effects [11, 24, 35, 36]. Consequently, the modulatory effect of KD and exogenous ketone supplements on the onset of isoflurane-generated anesthesia/immobility by not only adenosinergic pathways, but also other signaling pathways cannot be excluded. Moreover, delay in anesthesia induction evoked by KD and exogenous ketogenic supplements, which strongly correlated with the elevation of the blood βHB levels, suggest that nutritional ketosis may exert its effect on processes of anesthesia by delay of isoflurane anesthesia-evoked suppression of metabolic activity in neural networks [11]. Therefore, further studies are needed to reveal the exact mechanism of action of KD, and ketone supplements on isoflurane- (and other inhalational anesthetics) generated effects.

It has been demonstrated previously that WAG/Rij rats and G1D mice showed higher basal βHB levels compared to SPD rats and wild type mice, respectively [4, 21]. Moreover, G1D animals also showed βHB levels lower then WAG/Rij rats, while the increase in βHB levels after sub-chronic ketone supplementation (KE and KS) were higher in WAG/Rij rats compared to SPD rats. These results suggest that basal ketone levels and ketone supplements-evoked increase in blood βHB levels may be connected to either putative species-dependent differences or pathological conditions, such as epileptic seizures. However, in spite of different physiological (e.g., basal βHB level) and pathophysiological (e.g., spike-wave discharges/SWDs) conditions, KD and exogenous ketone supplementation generated similar delay in onset of isoflurane-generated anesthesia (immobility) in all of three rodent strains with (G1D mice and WAG/Rij rats) and without (SPD rats) pathology.

Our result suggests that the ketogenic diet and exogenous ketone supplementation-induced ketosis may have similar effects on the anesthetic response to isoflurane independent from the method of inducing ketosis, species studied (mice and rats) and presence (or lack of) of pathology. The ability of influencing the speed of inhalation induction may be important in clinical settings, for example, to delay induction in cyanotic children and in those with right-to-left shunts because in these patients the decreased pulmonary flow limits the rate of increase of the concentration of inhalational anesthetics in the arterial blood [37]. These results imply that the state of ketosis or these specific treatments may enhance neuroprotection from other harmful gases as well, therefore it is possible that achieving the state of ketosis might provide protection from toxic gases to first responders or military personnel, however further studies are needed to validate this hypothesis. Additional applications of these results could be considered and should be further explored. Nitrogen narcosis is a drowsy state induced by breathing air under high pressure in recreational or military divers producing anesthetic-like influence, resulting in temporarily impaired cognitive function, or even unconsciousness [38–41]. Since the mechanism of action in nitrogen narcosis is considered similar to anesthesia, achieving ketosis could also potentially delay its onset or mitigate negative consequences, but further studies are needed to confirm the potential effectiveness for such specific applications. Moreover, delaying the onset of anesthesia by nutritional ketosis or fasting ketosis may influence anesthetic procedures that surgeons and anesthesiologists need to consider prior to loss of consciousness for a medical procedure.

## Conclusion

The results of the present study suggest that KD- and exogenous ketone supplements-evoked nutritional ketosis can provide increased resistance to an anesthetic gas, such as isoflurane by delaying the onset of anesthesia. Further studies are warranted to determine the putative effect of ketogenic diet- and exogenous ketone supplements-evoked nutritional ketosis on other inhalational and intravenous anesthetics, as well as other harmful gases (such as carbon monoxide and volcanic gases) or chemical weapons/warfare-induced sleep-like effects [42, 43]. In addition, the ketosis induced influences on sleep-like effects will require further examination in human subjects, as such findings can have clinical and surgical relevance. Our results further suggest that it might be important to monitor blood ketone levels in humans undergoing inhalational anesthesia as this knowledge might prove helpful for the anesthesia provider. However, further studies are needed to reveal the exact neuropharmacological/metabolic mechanisms of KD and exogenous ketone supplementation on anesthetics-generated sleep-like effects and naturally occurring sleep.

## Abbreviations

A_1_R: adenosine A_1_ receptor
A_2A_R: adenosine A_2A_ receptor
ATP: Adenosine triphosphate
G1D: GLUT1 deficiency
GLUT1: glucose transporter 1
K_ATP_ channels: ATP-sensitive potassium channels
KD: ketogenic diet
KE: 1,3 butanediol-acetoacetate diester
KS: ketone salt
SD: standard diet
SPD rats: Sprague-Dawley rats
SWD: spike-wave discharge
WAG/Rij: Wistar Albino Glaxo/Rijswijk
βHB: beta-hydroxybutyrate

## References

[1] Stafstrom CE, Rho JM (2012). The ketogenic diet as a treatment paradigm for diverse neurological disorders. Front Pharmacol.

[2] D'Agostino DP, Pilla R, Held HE, Landon CS, Puchowicz M, Brunengraber H, Ari C, Arnold P, Dean JB (2013). Therapeutic ketosis with ketone ester delays central nervous system oxygen toxicity seizures in rats. Am J Physiol Regul Integr Comp Physiol.

[3] Poff AM, Ward N, Seyfried TN, Arnold P, D'Agostino DP (2015). Non-toxic metabolic management of metastatic cancer in VM mice: novel combination of ketogenic diet, ketone supplementation, and hyperbaric oxygen therapy. PLoS One.

[4] Ari C, Kovács Z, Juhasz G, Murdun C, Goldhagen CR, Koutnik AM, Poff AM, Kesl SL, D'Agostino DP (2016). Exogenous ketone supplements reduce anxiety-related behavior in Sprague-Dawley and Wistar albino Glaxo/Rijswijk rats. Front Mol Neurosci.

[5] Kesl SL, Poff AM, Ward NP, Fiorelli TN, Ari C, Van Putten AJ, Sherwood JW, Arnold P, D'Agostino DP (2016). Effects of exogenous ketone supplementation on blood ketone, glucose, triglyceride, and lipoprotein levels in Sprague-Dawley rats. Nutr Metab. Lond.

[6] Bostock EC, Kirkby KC, Taylor BV (2017). The current status of the ketogenic diet in psychiatry. Front Psychiatry.

[7] Kovács Z, D'Agostino DP, Dobolyi A, Ari C (2017). Adenosine A1 receptor antagonism abolished the anti-seizure effects of exogenous ketone supplementation in Wistar albino Glaxo Rijswijk rats. Front Mol Neurosci.

[8] Yudkoff M, Daikhin Y, Melø TM, Nissim I, Sonnewald U, Nissim I (2007). The ketogenic diet and brain metabolism of amino acids: relationship to the anticonvulsant effect. Annu Rev Nutr.

[9] Egan B, D'Agostino DP (2016). Fueling performance: ketones enter the mix. Cell Metab.

[10] Achanta LB (2017). Rae CD. β-Hydroxybutyrate in the brain: one molecule, multiple mechanisms. Neurochem Res.

[11] Constantinides C, Murphy K (2016). Molecular and integrative physiological effects of isoflurane anesthesia: the paradigm of cardiovascular studies in rodents using magnetic resonance imaging. Front Cardiovasc Med.

[12] Saper CB, Chou TC, Scammell TE (2001). The sleep switch: hypothalamic control of sleep and wakefulness. Trends Neurosci.

[13] Tung A, Mendelson WB (2004). Anesthesia and sleep. Sleep Med Rev.

[14] Moore JT, Chen J, Han B, Meng QC, Veasey SC, Beck SG, Kelz MB (2012). Direct activation of sleep-promoting VLPO neurons by volatile anesthetics contributes to anesthetic hypnosis. Curr Biol.

[15] Leung LS, Luo T, Ma J, Herrick I (2014). Brain areas that influence general anesthesia. Prog Neurobiol.

[16] Allen CN (2008). Circadian rhythms, diet, and neuronal excitability. Epilepsia.

[17] Masino SA, Kawamura M, Wasser CD, Pomeroy LT, Ruskin DN (2009). Adenosine, ketogenic diet and epilepsy: the emerging therapeutic relationship between metabolism and brain activity. Curr Neuropharmacol.

[18] Hallböök T, Ji S, Maudsley S, Martin B (2012). The effects of the ketogenic diet on behavior and cognition. Epilepsy Res.

[19] Hallböök T, Lundgren J, Rosén I (2007). Ketogenic diet improves sleep quality in children with therapy-resistant epilepsy. Epilepsia.

[20] Coenen AM, Van Luijtelaar EL (2003). Genetic animal models for absence epilepsy: a review of the WAG/Rij strain of rats. Behav Genet.

[21] Marin-Valencia I, Good LB, Ma Q, Duarte J, Bottiglieri T, Sinton CM, Heilig CW, Pascual JM (2012). Glut1 deficiency (G1D): epilepsy and metabolic dysfunction in a mouse model of the most common human phenotype. Neurobiol Dis.

[22] Hemmings HC, Akabas MH, Goldstein PA, Trudell JR, Orser BA, Harrison NL (2005). Emerging molecular mechanisms of general anesthetic action. Trends Pharmacol Sci.

[23] Sonner JM, Antognini JF, Dutton RC, Flood P, Gray AT, Harris RA, Homanics GE, Kendig J, Orser B, Raines DE, Rampil IJ, Trudell J, Vissel B, Eger EI (2003). Inhaled anesthetics and immobility: mechanisms, mysteries, and minimum alveolar anesthetic concentration. Anesth Analg.

[24] Rogawski MA, Löscher W, Rho JM. Mechanisms of action of antiseizure drugs and the ketogenic diet. Cold Spring Harb Perspect Med 2016;6(5). pii: a022780. 10.1101/cshperspect.a022780 PMC4852797 26801895

[25] Patel AJ, Honoré E (2001). Anesthetic-sensitive 2P domain K+ channels. Anesthesiology.

[26] Sirois JE, Lynch C, Bayliss DA (2002). Convergent and reciprocal modulation of a leak K+ current and I(h) by an inhalational anaesthetic and neurotransmitters in rat brainstem motoneurones. J Physiol.

[27] Eikermann M, Vetrivelan R, Grosse-Sundrup M, Henry ME, Hoffmann U, Yokota S, Saper CB, Chamberlin NL (2011). The ventrolateral preoptic nucleus is not required for isoflurane general anesthesia. Brain Res.

[28] Hawkins RA, Biebuyck JF (1979). Ketone bodies are selectively used by individual brain regions. Science.

[29] Kovács Z, Juhász G, Palkovits M, Dobolyi A, Kékesi KA (2011). Area, age and gender dependence of the nucleoside system in the brain: a review of current literature. Curr Top Med Chem.

[30] Porkka-Heiskanen T, Strecker RE, Thakkar M, Bjorkum AA, Greene RW, McCarley RW (1997). Adenosine: a mediator of the sleep-inducing effects of prolonged wakefulness. Science.

[31] Roald OK, Forsman M, Steen PA (1990). Partial reversal of the cerebral effects of isoflurane in the dog by theophylline. Acta Anaesthesiol Scand.

[32] Chamberlin NL, Arrigoni E, Chou TC, Scammell TE, Greene RW, Saper CB (2003). Effects of adenosine on gabaergic synaptic inputs to identified ventrolateral preoptic neurons. Neuroscience.

[33] Brambilla D, Chapman D, Greene R (2005). Adenosine mediation of presynaptic feedback inhibition of glutamate release. Neuron.

[34] Huang ZL, Urade Y, Hayaishi O (2011). The role of adenosine in the regulation of sleep. Curr Top Med Chem.

[35] Kofke WA, Hawkins RA, Davis DW, Biebuyck JF (1987). Comparison of the effects of volatile anesthetics on brain glucose metabolism in rats. Anesthesiology.

[36] Joksovic PM, Weiergräber M, Lee W, Struck H, Schneider T, Todorovic SM (2009). Isoflurane-sensitive presynaptic R-type calcium channels contribute to inhibitory synaptic transmission in the rat thalamus. J Neurosci.

[37] Miller-Hance WC. Anesthesia for noncardiac surgery in children with congenital heart disease. In: Cote C, Lerman J, Todres ID, editors. A practice of anesthesia for infants and children. Fourth ed. Philadelphia: Saunders-Elsevier; 2009.

[38] Leitch DR (1971). Medical aspects of a simulated dive to 1,500 feet (458 metres). Proc R Soc Med.

[39] Bennett PB, McLeod M (1984). Probing the limits of human deep diving. Philos Trans R Soc Lond Ser B Biol Sci.

[40] Edmonds C, McKenzie B, Thomas R. Unconsciousness in divers. In: diving medicine for scuba divers 2013. 5th Edition; Free Internet Edition; http://www.divingmedicine.info/Book%20DMfSD%202013.pdf. Accessed 10 Oct 2017.

[41] Bove AA (2014). Diving medicine. Am J Respir Crit Care Med.

[42] Baxter PJ, Kapila M, Mfonfu D (1989). Lake Nyos disaster, Cameroon, 1986: the medical effects of large scale emission of carbon dioxide?. Brit Med J.

[43] Romano JA, Lukey BJ, Salem H (2008). Chemical warfare agents chemistry, pharmacology, toxicology, and therapeutics.

